# Massive migration promotes the early spread of COVID-19 in China: a study based on a scale-free network

**DOI:** 10.1186/s40249-020-00722-2

**Published:** 2020-08-10

**Authors:** Wen-Yu Song, Pan Zang, Zhong-Xing Ding, Xin-Yu Fang, Li-Guo Zhu, Ya Zhu, Chang-Jun Bao, Feng Chen, Ming Wu, Zhi-Hang Peng

**Affiliations:** 1 School of Pediatrics, Nanjing Medical University, Nanjing, 211166 Jiangsu China; 2 Department of Epidemiology and Biostatistics, School of Public Health, Nanjing Medical University, Nanjing, 211166 Jiangsu China; 3 Center for Global Health, School of Public Health, Nanjing Medical University, Nanjing, 211166 Jiangsu China; 4 Jiangsu Provincial Center for Disease Control and Prevention, Nanjing, 211166 Jiangsu China; 5 Institude of Healthy Jiangsu Development, Nanjing Medical University, Nanjing, 211166 Jiangsu China

**Keywords:** COVID-19, Migration, Scale-free network

## Abstract

**Background:**

The coronavirus disease 2019 (COVID-19) epidemic met coincidentally with massive migration before Lunar New Year in China in early 2020. This study is to investigate the relationship between the massive migration and the coronavirus disease 2019 (COVID-19) epidemic in China.

**Methods:**

The epidemic data between January 25th and February 15th and migration data between Jan 1st and Jan 24th were collected from the official websites. Using the *R* package *WGCNA*, we established a scale-free network of the selected cities. Correlation analysis was applied to describe the correlation between the Spring Migration and COVID-19 epidemic.

**Results:**

The epidemic seriousness in Hubei (except the city of Wuhan) was closely correlated with the migration from Wuhan between January 10 and January 24, 2020. The epidemic seriousness in the other provinces, municipalities and autonomous regions was largely affected by the immigration from Wuhan. By establishing a scale-free network of the regions, we divided the regions into two modules. The regions in the brown module consisted of three municipalities, nine provincial capitals and other 12 cities. The COVID-19 epidemics in these regions were more likely to be aggravated by migration.

**Conclusions:**

The migration from Wuhan could partly explain the epidemic seriousness in Hubei Province and other regions. The scale-free network we have established can better evaluate the epidemic. Three municipalities (Beijing, Shanghai and Tianjin), eight provincial capitals (including Nanjing, Changsha et al.) and 12 other cities (including Qingdao, Zhongshan, Shenzhen et al.) were hub cities in the spread of COVID-19 in China.

## Background

The coronavirus disease 2019 (COVID-19) is a contagion with strong infectivity [1]. Evidence has proved that COVID-19 can be transmitted from the wildlife to human beings [2]. COVID-19 has been spreading around China from early 2020. Migration may play a key role in the spread of COVID-19 [3]. Here, we proved that migration from Wuhan before the lunar New Year promoted the wide spread of the epidemic. However, the migration from Wuhan could only partly explain the wild spread of COVID-19 in China. The relation between the whole Spring Migration and the COVID-19 epidemic was further explored in this study.

Scale-free network is a classic model of complex networks [4]. The concept was first put forward in a world wide web investigation by an internet research team in 1999 [5]. Currently, the scale-free network is widely used in epidemiology [6], sociology [7] and genomics [8]. A good example of its application is the weighted gene co-expression network analysis (WGCNA), which can tease out the relationship between thousands of genes in a biological process [9]. In WGCNA, genes with different expression patterns in the collected samples can be divided by the scale-free network [10].

The epidemic synchronized the massive migration before Lunar New Year in China and thus the specific relationship between the massive migration and COVID-19 epidemic in China was further investigated by the scale-free network in the present study. We collected all regions reporting confirmed COVID-19 cases in China between 1st January and 15th February. We further described the correlation between the migration and the epidemic. The cities were clustered into two different modules in a scale-free network. The epidemics in the brown module were highly correlated with the migration. The migration in these cities should be strictly monitored to control the spread of COVID-19.

## Methods

### Data

Retrospective study was designed to progress the research. Migration data between January 1st and February 24th. were collected from Location Baidu Service (LBS, http://qianxi.baidu.com/). Epidemic data were acquired from National Health Commission of the People’s Republic of China (http://www.nhc.gov.cn/) and health commission of local governments.

### Plotting of epidemic maps

The vector data of the map of China and Guangdong, Zhejiang was downloaded from *National Geomatics Center of China* (*http://www.ngcc.cn/ngcc/*). The R (*3.6.3, AT&T BellLaboratories, New Jersey, USA*) package *rgdal 3.3.1* was used to read the *shp* file. R package *ggplot2 1.5–10* was further used to plot the outline of the map. *Adobe Illustrator (CC2019, Adobe Systems Incorporated, California, USA)*, a vector graph editing software, was applied to add the epidemic data on the map.

### Establishment of scale-free network

R package *WGCNA* (*1.69, University of California, California, USA*) was used to establish the scale-free network [9]. *PickSoftThreshold, TOMsimilarity, cutreeDynamic,* three main functions in WGCNA, were applied in further analysis. *PickSoftThreshold* was employed to pick the soft threshold and *TOMsimilarity* was used to acquire the *TOM* matrix. We clustered the regions into different modules with *cutreeDynamic*. In *WGCNA*, eigengene is a fictitious gene to describe the characteristic expression pattern of the genes in a module. Here, we clustered the regions with similar migration patterns into a module, which has the same meaning to the eigengene in WGCNA. *Cytoscape (3.7.1, National Institute of General Medical Sciences, Maryland, USA,* *https://cytoscape.org/**)* was applied to draw the network of the regions according to the calculated relationships between the regions.

### Correlation analysis

Pearson correlation analysis by *R* was conducted to evaluate the relationship between the number of migrants and the total number of confirmed cases. *P <* 0.05 was considered statistically significant.

## Results

### Migration from Wuhan between January 10 and January 24 ignited the epidemic of COVID-19 in China

**Fig. 1:**
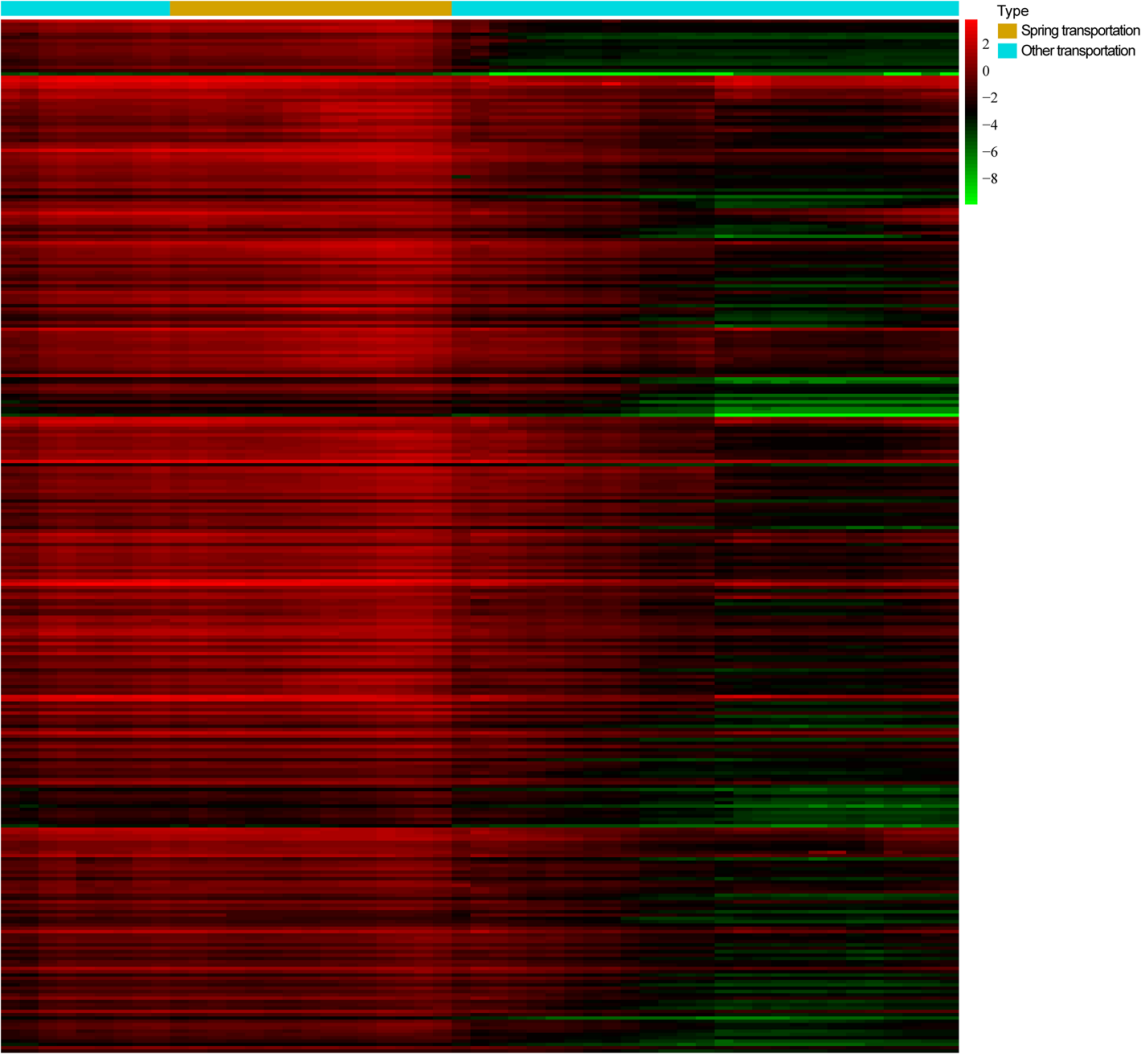
Correlation between the number of migrants from Wuhan between January 10 and January 24, 2020 and the confirmed cases between Jan 25 and Feb 15. **a** Correlation between the number of migrants and the cases in all regions of Hubei Province except Shennongjialin region (migration data not shown). **b** Correlation between the number of migrants and the cases in 31 provinces of China (except Taiwan, Hong Kong and Macao, migration data not shown) (**c**) Epidemic situation in Zhejiang on February 20, 2020 (**d**) Epidemic situation in Guangdong on February 20, 2020. Dotted lines represent 90% prediction band

Correlation analysis was conducted to describe the relationship between the epidemic seriousness and the migration into the other regions except Wuhan in Hubei (*R*^*2*^ *=* 0.9300*, P <* 0.0001). All the points in the plot were in the range of the 90% prediction band. Similarly, the epidemic situations in the other provincial regions except Hubei were significantly correlated with the migration from Wuhan (*R*^*2*^ *=* 0.6556*, P <* 0.0001). Among these regions, Guangdong and Zhejiang were out of the 90% prediction band. Guangdong showed the second largest number of confirmed cases, and Zhejiang with the fourth. The epidemic situations in the two provinces were demonstrated in Fig. 1c and d. Wenzhou (ZJ), Shenzhen (GD) and Guangzhou (GD) were the three cities with the largest number of confirmed cases in both provinces.

### Migration between January 1, 2020 and February 20, 2020

**Fig. 2:**
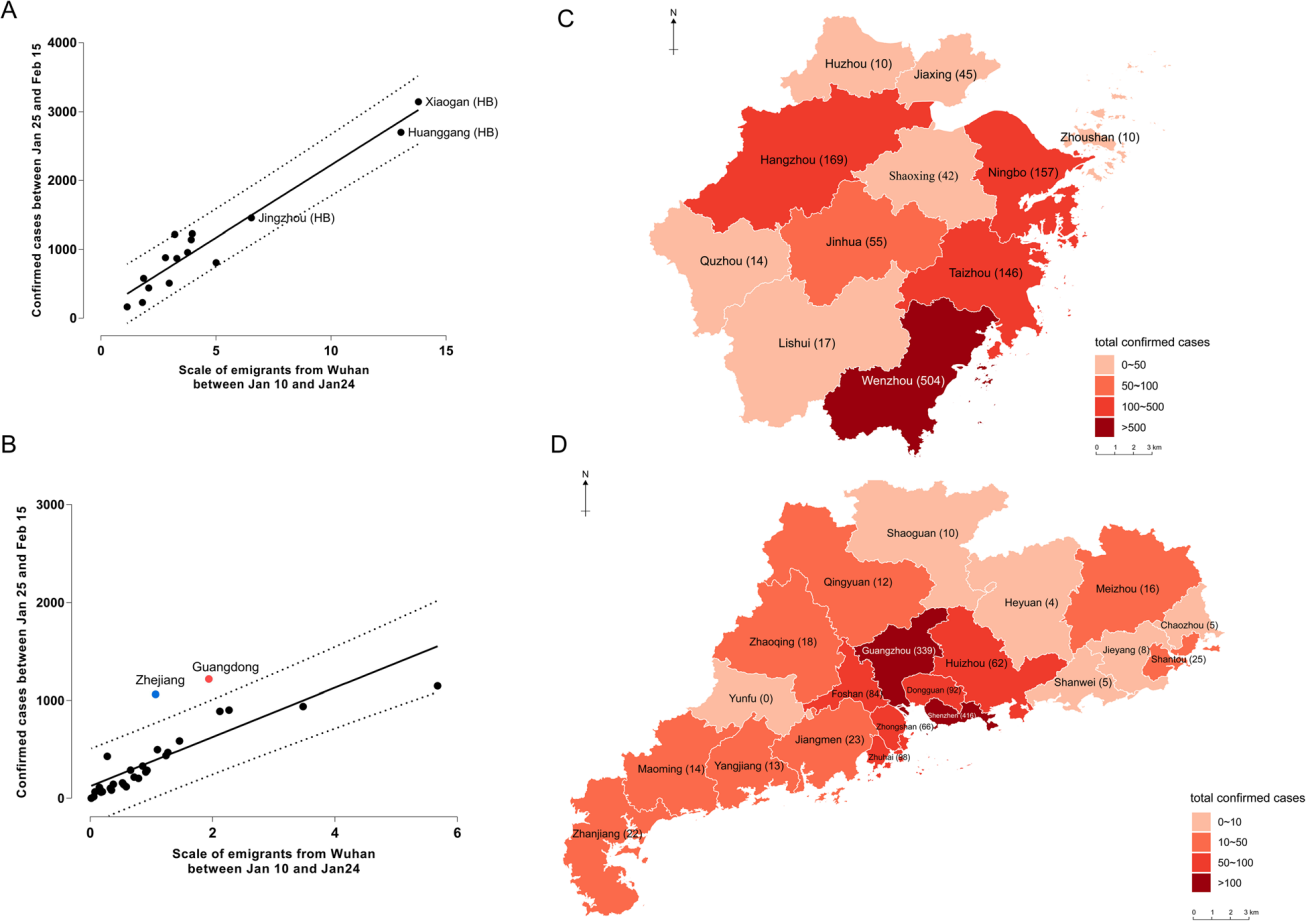
Scales of migration of COVID-19-epidemic regions in China between Jan 1, 2020 and Feb 20, 2020. Migration data were log2-calculated; Deeper red represents larger scale of migration

The migration data in 296 regions with confirmed cases was collected and visualized as a heatmap (Fig. 2). The data was treated with *log*_*2*_ algorithm to make the heatmap more readable. Red represented larger scale of migration and green represented the contrary. During the migration from January 10 to January 24, the migration remained at a high level. After the lunar New Year (January 25), the migration was blocked by the government to control the spread of epidemic. More details were shown in the Additional Files 1 “Migration data between Jan,10 and Jan,24.xlsx”.

### Modules in the scale-free network

**Fig. 3:**
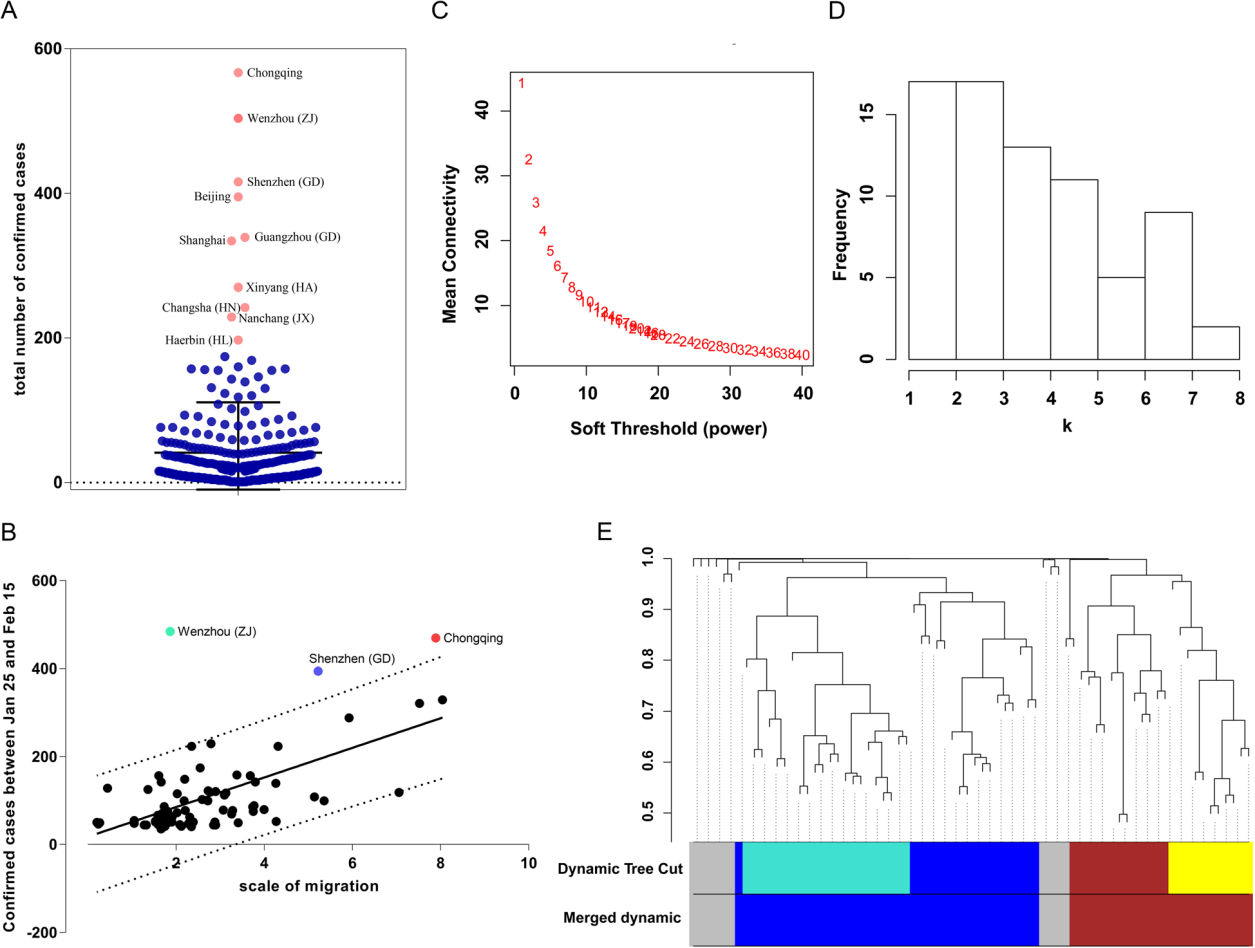
Clusters of the selected city by the scale of migration. **a** Epidemic situation in the reported cities (**b**) Correlation of the scale of migration and confirmed cases between Jan 25 and Feb 15 (**c**) Mean connectivity by the potential soft thresholds (**d**) Confirmation of the selected soft threshold (**e**) Clustering of the top 25% of cities which had the highest number of confirmed cases by WGCNA. Dotted lines represent 90% prediction band

The epidemic data was plotted in Fig. 3a. The distribution was skewed and the top 10 epidemic-inflicted cities were labeled in light red. The correlation analysis showed the relationship between the scale of migration and the confirmed cases between Jan 25 and Feb 15 (*R*^*2*^ *=* 0.3449*, P <* 0.0001*,* Fig. 3b). Top 25% cities (76 cities) were chosen for further investigation. Soft threshold was picked to be 38 (Fig. 3c) and the verification of the soft threshold was displayed in Fig. 3d. The regions were clustered into two modules: blue and brown (Fig. 3e). Those cities which could not be clustered into either of both were labeled grey, according to the *WGCNA* package [9].

### Correlation between the immigration and the epidemic in the selected two modules

**Fig. 4:**
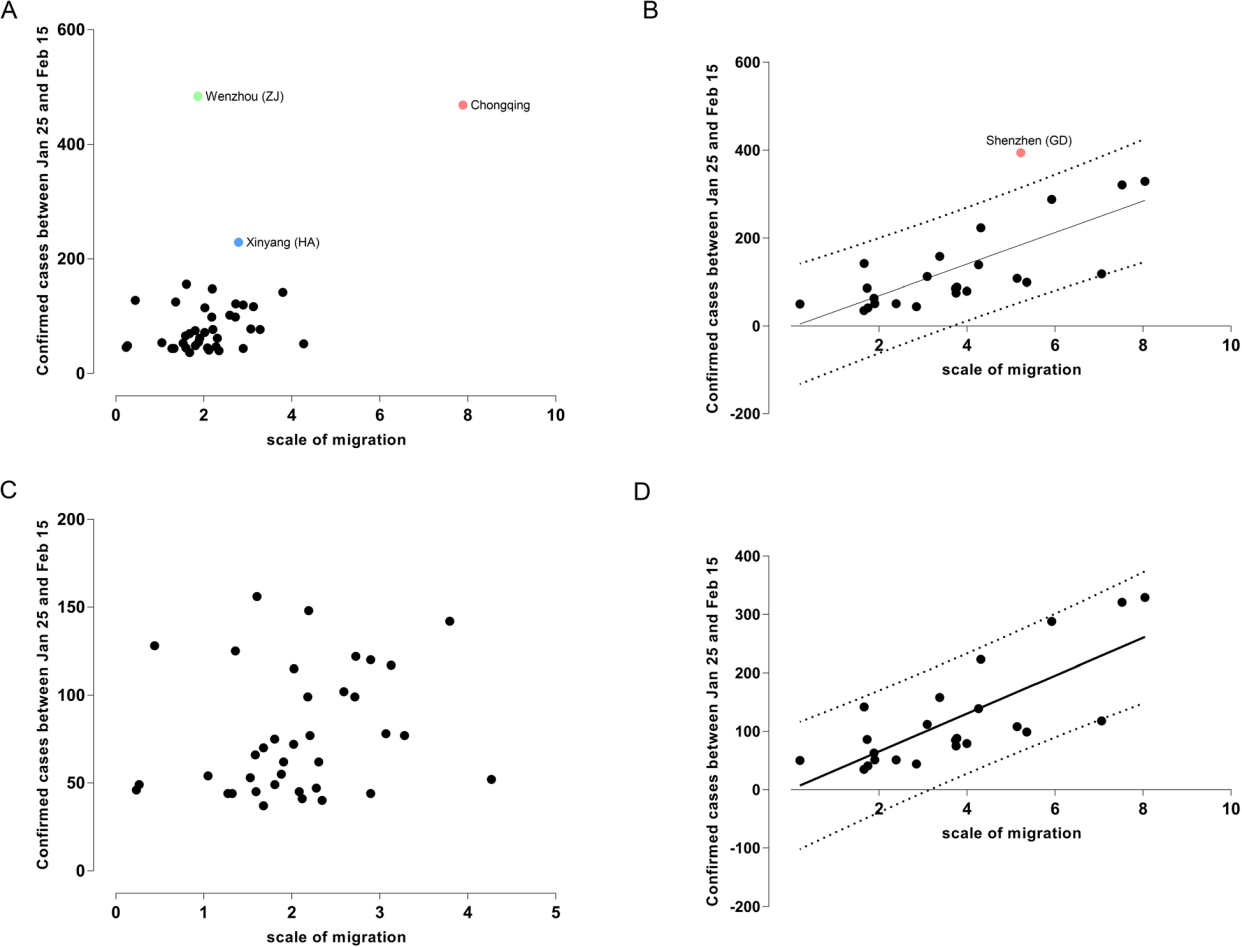
Correlation between the migration scale and the confirmed cases between Jan 25 and Feb 15 in blue and brown modules. **a** Correlation between the migration scale and the cases in blue module (**b**) Correlation between the migration scale and the in brown module (**c**) Correlation between the migration scale and the cases in blue module except Chongqing, Wenzhou (ZJ) and Xinyang (HA) (**d**) Correlation between the migration scale and the cases in the brown module except Shenzhen (GD). Dotted lines represent 90% prediction band

The distribution of the regions in the blue module was plotted in Fig. 4a and the correlation analysis showed a significant correlation between the immigration and the epidemic in the blue module (*R*^*2*^ *=* 0.2615*, P =* 0.0007). Similarly, the epidemic situation was significantly correlated with the immigration in the brown module (*R*^*2*^ *=* 0.5071*, P <* 0.0001). However, Wenzhou (ZJ), Xinyang (HA) and Chongqing were obvious outliers. After these three cities were excluded, no correlation was observed between the migration and the epidemic in the blue module. Instead, the correlation *R*^*2*^ reached a higher value of 0.5765 after deletion of Shenzhen (GD).

### Network of the regions in the brown module

**Fig. 5:**
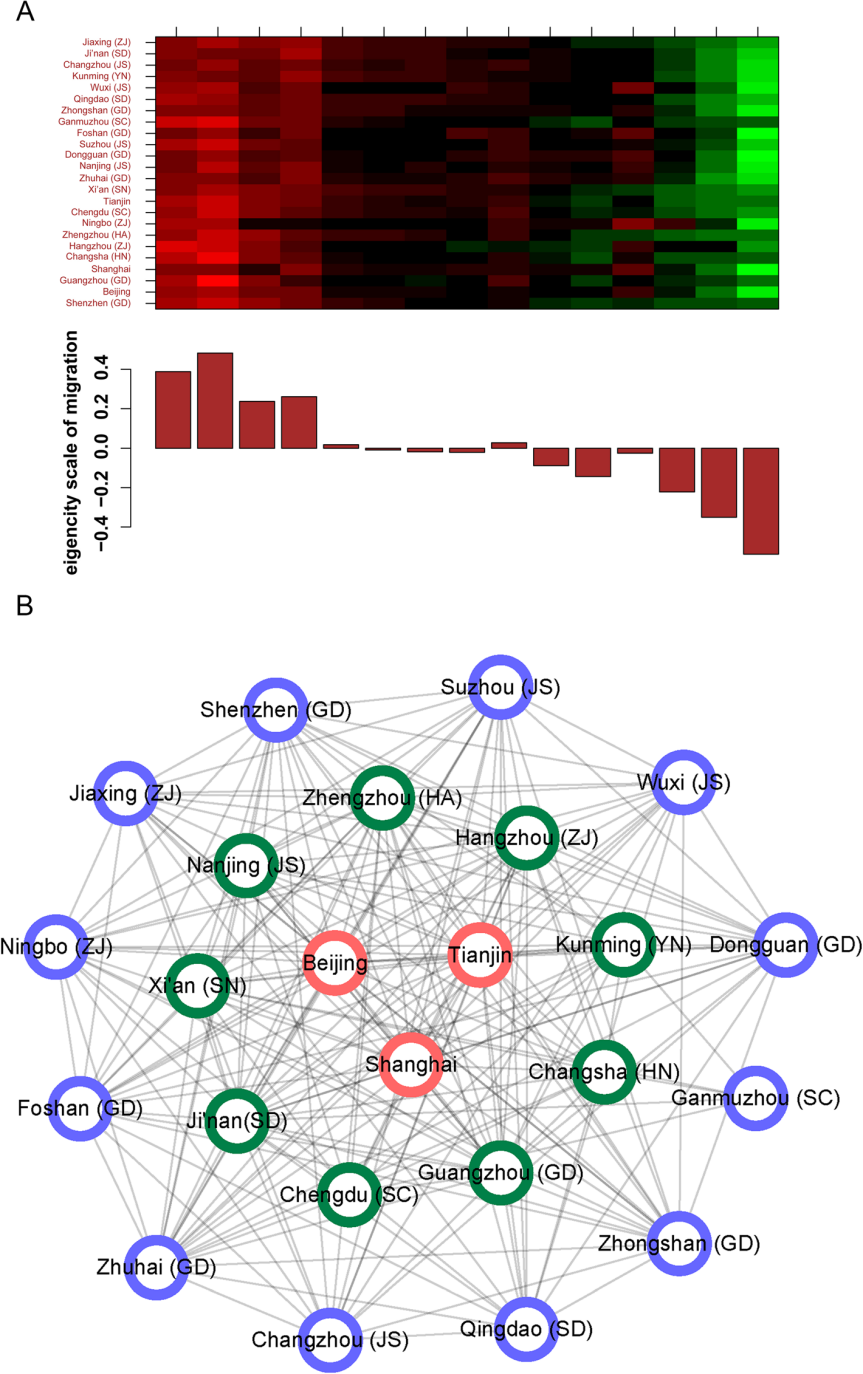
Network of the cities in the brown module. **a** Migration characteristic of the cities in the brown module (**b**) Network of the cities in the brown module. Migration data were log2-calculated; deeper red represents larger scale of migration. Municipalities are labeled as red, provincial capitals as green and other cities as blue

The migration data of the 24 cities in the brown module between January 10 and January 24 was displayed in Fig. 5a. The immigrants gradually decreased from January 10 to January 24. The network of the 24 regions was established in Fig. 5b. Municipalities were labeled with red, provincial capitals with green and other cities with blue. Three municipalities (Beijing, Shanghai and Tianjin), eight provincial capitals (including Nanjing, Changsha et al.) and 12 other cities (including Qingdao, Zhongshan, Shenzhen et al.) were included in the network.

## Discussion

COVID-19 epidemic spreads erupts in Wuhan, and in Hubei and China over the past 2 months [11]. Large-scale and intense migration (as shown by the heavy transportation) on the eve of the lunar New Year may accelerate the spread of the disease [12]. In the present study, we collected the data on the immigrants into other cities from Wuhan between January 10 and January 24. Correlation analysis was conducted to evaluate the relationship between the outflow from Wuhan and the confirmed cases between January 25 and February 15 in other cities, in the condition that the incubation time of COVID-19 was 14 days [13]. It was clear that the number of migrants from Wuhan was significantly correlated with the epidemic seriousness in other regions of Hubei province (*R*^*2*^ *=* 0.9300*, P <* 0.0001), with all the points in the 90% prediction band (Fig. 1a). This correlation was also found in other provinces, municipalities and autonomous regions (Fig. 1b*, R*^*2*^ *=* 0.6556*, P <* 0.0001). Among all the regions, Zhejiang and Guangdong were out of the 90% prediction band, showing more confirmed cases than others. By displaying the map of Zhejiang and Guangdong (Fig. 1c and d), we further analyzed the confirmed cases in all the prefecture-level cities in these two provinces. Wenzhou (ZJ) (504 confirmed cases), Shenzhen (GD) (416 confirmed cases) and Guangzhou (GD) (339 confirmed cases) were the three with the most serious epidemics.

However, the spread of the epidemic is a complex process. The seriousness of the epidemic cannot be measured only by the migration from Wuhan. In addition, useful information about the epidemic could be lost if our correlation analysis was conducted on the basis of provincial data. Different levels of cities in the same province might display more details about the migration. Therefore, we collected all the migrant data in the COVID-19 epidemic regions between January 1 and February 20. From the heatmap, we found that during the Migration from January 10 to January 24, the number of migrants reached a peak (Fig. 3), and dropped after the stringent controlling of the government. According to the total number of confirmed cases by February 20, we found a skewed distribution of the numbers in these regions (Fig. 3a). Chongqing, Wenzhou (ZJ) and Shenzhen (GD) were the three cities with the most obvious change.

Similarly, we analyzed the correlation between the scale of migration and the confirmed cases between January 25 and February 15 (Fig. 3b*, R*^*2*^ *= 0.3449, P <* 0.0001). Although the correlation was statistically significant, we did not consider the model showed a good fitness (0.3449). The outcome suggested that it was worthwhile to further investigate the relationship between migration and epidemic by dividing the regions into different modules by their characteristics. In our study, we predicted that the cities with varying confirmed cases in China during the Spring Migration can be analyzed by a scale-free network. Since the government initiated strict controlling efforts after the lunar New Year, our scale-free network incorporated the migration data between January 10 and January 24 in the top serious 25% epidemic cities out of Hubei. Thirty-eight was chosen as the soft threshold (Fig. 3c and d). By calculating the TOM matrix, the selected cities were clustered into two modules.

In addition, by plotting the number of immigrants and the confirmed cases between Jan 25 and Feb 15 in the 52 cities in the blue module (Fig. 4a), we screened out three outliers, Chongqing, Wenzhou (ZJ), and Xinyang (HA). Similarly, correlation analysis was conducted in the brown module (*R*^*2*^ *=* 0.5071*, P <* 0.0001), with Shenzhen (GD) out of the 90% prediction (Fig. 4b). Notably, after removing Chongqing, Wenzhou (ZJ), Xinyang (HA) and Shenzhen (GD) from the plots, we found no correlation in the blue module (Fig. 4c*, R*^*2*^ *=* 0.0495, *P =* 0.1855) and a higher fitness in the brown module (Fig. 4d*, R*^*2*^ *=* 0.5765*, P <* 0.0001). The results showed that the cities in the brown module might play a decisive rule in the spread of the epidemic.

Therefore, we analyzed the brown module consisting of 24 cities: three municipalities, nine provincial capitals, and the left 12 cities including Dongguan (GD), Qingdao (SD), Ningbo (ZJ), Suzhou (JS) that are all trade centers and transportation hubs. The immigration tendencies were described in Fig. 5a. In addition, we established the network. As shown in Fig. 5b, of the 24 cities, five are in Guangdong and three in Jiangsu, suggesting that these two provinces should take stricter management to control the spread of COVID-19 epidemic. Thus, we considered the epidemics in the cities in this brown module may decide the whole situation. The related governments must curb the migration into the region. In the blue module, Chongqing, Wenzhou (ZJ) and Xinyang (HA) were regarded special. Chongqing neighbors Hubei. Xinyang (HA) is near to Hubei. Therefore, we considered the short geographical distance and the convenient transportation caused the epidemics in both cities. As for Wenzhou (ZJ), the serious epidemic might result from the businessmen rushing back from Wuhan before the lunar New Year.

The main limitation of the study is the data volume. In conventional *WGCNA*, thousands of genes with different expression patterns in a biological process were applied to establish the scale-free network [9]. However, the number of cities in China is far less than the count of genes in cells. In this study, only 76 cities were introduced for the establishment of the scale-free network. The network showed a benign fitness of epidemic and successfully screened out the key cities from the selected 76 cities. Thus, the scale-free network can successfully explain the epidemic of COVID-19 in China to a certain extent. Additionally, we did not divide the COVID-19 cases of the cities into imported and secondary cases, as the migration would possibly lead to imported cases outside Hubei. Epidemiological bias might happen.

## Conclusions

The migration from Wuhan could partly explain the epidemic situation in other regions. Migration between some major cities plays a crucial role in the spread of COVID-19. Related governments should take strict efforts to reduce the migration and control the continuing spread of COVID-19.

## Additional File

**Additional file 1.** Migration data between Jan, 10 and Jan, 24.

## Data Availability

All data generated or analyzed supporting the findings of this article are included within the article and its additional files.

## Abbreviations

COVID-19: The coronavirus disease
WGCNA: Weighted gene co-expression network analysis
HB: Hubei
HN: Hunan
JX: Jiangxi
JS: Jiangsu
SD: Shandong
AH: Anhui
HL: Heilongjiang
JL: Jilin
LN: Liaoning
GS: Gansu
QH: Qinghai
XJ: Xinjiang
XZ: Xizang
SC: Sichuan
ZJ: Zhejiang
FJ: Fujian
GD: Guangdong
HI: Hainan
TW: Taiwan
SX: Shanxi
SN: Shaanxi
HE: Hebei
HA: Henan
NX: Ningxia
NM: Inner Mongolia
YN: Yunnan
BJ: Beijing
TJ: Tianjin
CQ: Chongqing
HK: Hong Kong
MO: Macao
GX: Guangxi
GZ: Guizhou
Municipalities: Beijing, Tianjin, Chongqing, Shanghai (four cities under the direct control of the central government)
